# Mechanism, Formulation, and Efficacy Evaluation of Natural Products for Skin Pigmentation Treatment

**DOI:** 10.3390/pharmaceutics16081022

**Published:** 2024-08-01

**Authors:** Xueli Peng, Yuning Ma, Chenxin Yan, Xiaocen Wei, Linlin Zhang, Hehe Jiang, Yuxia Ma, Suohui Zhang, Mengzhen Xing, Yunhua Gao

**Affiliations:** 1 Qingdao Academy of Chinese Medical Sciences, Shandong University of Traditional Chinese Medicine, Qingdao 266112, China; 2022111558@sdutcm.edu.cn; 2 Key Laboratory of New Material Research Institute, Institute of Pharmacy, Shandong University of Traditional Chinese Medicine, Jinan 250355, China; 60210001@sdutcm.edu.cn (Y.M.); 60050072@sdutcm.edu.cn (X.W.); linlin66210@outlook.com (L.Z.); 18396852678@163.com (H.J.); 3 Beijing CAS Microneedle Technology, Ltd., Beijing 102609, China; yanchenxin@casmn.com; 4 Department of Acupuncture-Moxibustion and Tuina, Shandong University of Traditional Chinese Medicine, Jinan 250355, China; 60050012@sdutcm.edu.cn; 5 Key Laboratory of Photochemical Conversion and Optoelectronic Materials, Technical Institute of Physics and Chemistry of Chinese Academy of Sciences, Beijing 100190, China; suohuizhang@mail.ipc.ac.cn

**Keywords:** skin pigmentation, natural products, mechanism, novel formulation, pharmacodynamic evaluation

## Abstract

Skin pigmentation typically arises from the excessive secretion and accumulation of melanin, resulting in a darker complexion compared to normal skin. Currently, the local application of chemical drugs is a first-line strategy for pigmentation disorders, but the safety and efficacy of drugs still cannot meet clinical treatment needs. For long-term and safe medication, researchers have paid attention to natural products with higher biocompatibility. This article begins by examining the pathogenesis and treatment approaches of skin pigmentation diseases and summarizes the research progress and mechanism of natural products with lightening or whitening effects that are clinically common or experimentally proven. Moreover, we outline the novel formulations of natural products in treating pigmentation disorders, including liposomes, nanoparticles, microemulsions, microneedles, and tocosomes. Finally, the pharmacodynamic evaluation methods in the study of pigmentation disorder were first systematically analyzed. In brief, this review aims to collect natural products for skin pigmentation treatment and investigate their formulation design and efficacy evaluation to provide insights for the development of new products for this complex skin disease.

## 1. Introduction

Skin pigmentation manifests as uneven brown to dark brown spots on the skin [1,2]. The onset of skin pigmentation is related to factors such as ultraviolet radiation, hormonal changes, cellular inflammation, skin damage, acne, and drug effects [3,4]. Influenced by various factors, melanocytes undergo excessive proliferation, aggregation, and secretion of pigment, resulting in the formation of pigmentation and freckles [1]. At present, topical chemical agents, including hydroquinone, kojic acid, retinoic acid, azelaic acid, niacinamide, vitamin C, and vitamin E, are first-line therapies for the clinical treatment of pigmentation diseases [5]. However, most of these substances have drawbacks such as cytotoxicity, irritation, instability, or unsatisfactory therapeutic effects, which may lead to side effects and poor patient compliance.

In recent years, natural products with spot-lightening and whitening effects have garnered increasing attention due to their natural, non-toxic, and excellent biocompatible characteristics [6]. Additionally, the safety and efficacy of these products have been widely confirmed through cytotoxicity, tyrosinase activity [7], antioxidant levels [8], melanogenesis inhibition [9], and the established animal models of pigmentation [10,11]. Meanwhile, novel delivery systems, including liposomes [12], nanoparticles [13], nanoemulsions [14], and microneedles [15], have been utilized to improve drug solubility, increase skin permeability or reduce skin irritation, thereby enhancing therapeutic effects [16]. Undoubtedly, the study of natural products for pigmentation treatment presents excellent potential and value, both in terms of creating social and economic benefits.

In this review, we focus on the utilization and mechanism of natural products in treating skin pigmentation, considering the pathogenesis and therapeutic methods of diseases. Furthermore, the novel formulations of natural products applied in skin pigmentation treatment are summarized. Finally, the classical pharmacodynamic evaluation methods in studying pigmentation disorders are highlighted to provide a reference for developing new natural product formulations.

## 2. Pathogenesis and Therapeutic Approaches

### 2.1. Pathogenesis

The skin is the body’s largest organ, comprising three main layers: epidermis, dermis, and subcutaneous fat layer. It also contains numerous nerves, nerve endings, glands, hair follicles, and blood vessels beneath its surface [17]. Melanocytes, which are responsible for the color of the epidermal layer, originate embryologically from neural crest cells. These melanocytes are distributed throughout the basal layer of the epidermis and produce melanin, the primary determinant of skin color [18]. Pigmentation diseases are caused by the excessive accumulation or abnormal distribution of melanin in the skin. Melanin, a natural pigment that gives color to our skin, hair, and eyes, is responsible for these conditions. Under normal circumstances, the production, distribution, and metabolism of melanin are balanced. However, certain diseases or pathological conditions can disrupt this balance, resulting in abnormal melanin deposition and leading to hyperpigmentation disorders [3].

**Figure 1:**
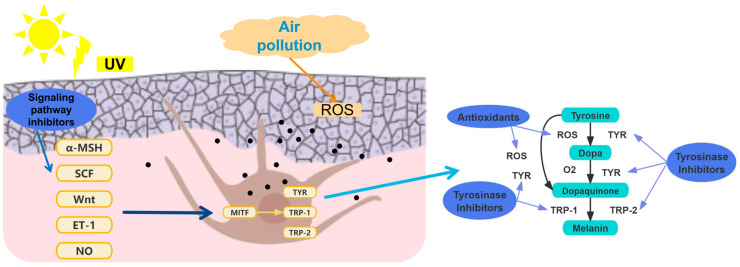
The pathogenesis and therapeutic approaches of skin pigmentation.

The biosynthesis pathway of melanin is a complex process involving a series of enzymatic and chemical reactions [19]. In melanocytes, phenylalanine is first converted to L-tyrosine, which is then oxidized to L-dopa by the action of tyrosinase (TYR). L-dopa is further oxidized to dopaquinone, a key step in melanin synthesis. Dopaquinone undergoes oxidation-reduction reactions to form dopachrome. Afterwards, it is decarboxylated to 5,6-dihydroxyindole or converted to 5,6-dihydroxyindole-2-carboxylic acid with the help of tyrosinase-related protein-2 (TRP-2). These intermediates are subsequently transformed into ketoindoles by tyrosinase-related protein-1 (TRP-1). Ultimately, melanin formation is transferred to melanosomes. After the melanosome matures, melanin is transferred from melanosomes to adjacent keratinocytes via dendrites [20,21]. The keratinocytes internalize melanosomes and migrate melanin to the peripheral membrane region, leading to melanin accumulation and causing skin pigmentation [22,23,24], as shown in Figure 1.

Simultaneously, multiple signaling pathways are involved in regulating melanin formation, including α-melanocyte stimulating hormone (α-MSH)-induced signaling pathway, PI3K/Akt signaling pathway, SCF/c-kit-mediated MAPK signaling pathway, Wnt/β-catenin signaling pathway, NO/cGMP signaling pathway, cytokine, transcription factor PAX3, and liver X receptor-mediated signaling pathway [25]. Although various cellular signals are transmitted through different signaling pathways, most of them converge to the microphthalmic aberrant transcription factor (MITF), making MITF a transcription factor that integrates upstream signals and regulates downstream genes, thereby regulating melanin biosynthesis [25].

### 2.2. Therapeutic Approaches

Skin pigmentation treatment is extremely challenging due to the complexity of its etiology and pathogenesis. The common therapies involve the application of physical sunscreen alone or in combination with topical decolorizing agents, such as hydroquinone, kojic acid, retinoic acid, and azelaic acid [26]. They aim to exert therapeutic effects by reducing melanin synthesis or transfer, attenuating oxidative stress response, repairing the skin barrier, and improving microcirculation [27]. Among them, inhibiting melanin biosynthesis and combating oxidative stress are the most frequently employed strategies. Tyrosinase and tyrosine-related proteins (TRP-1, TRP-2) play crucial roles in the catalytic pathway of melanin synthesis, serving as rate-limiting steps [28]. Consequently, inhibiting tyrosinase activity can effectively curtail melanin production. In addition, the overexpression of reactive oxygen species (ROS) free radicals in the skin under oxidative stress stimuli significantly contributes to pigmentation exacerbation [29]. ROS acts on the skin’s basal layer and promotes the accelerated secretion of melanin by melanocytes, thereby causing skin pigmentation disorders. Hence, antioxidants represent another efficacious route to impede melanin deposition (Figure 1) [30,31,32].

## 3. Natural Products for Treating Skin Pigmentation

### 3.1. Natural Medicinal Ingredients

**Figure 2:**
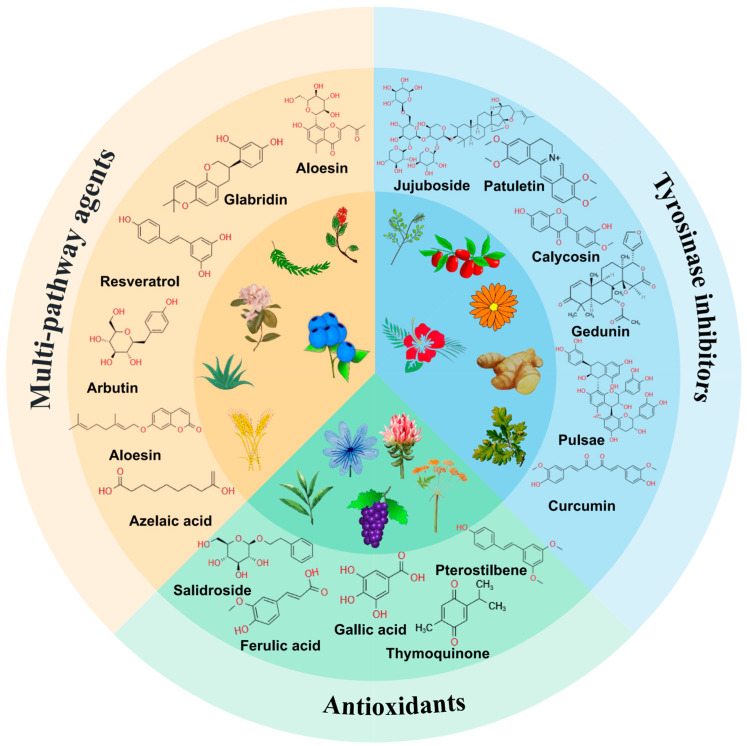
The structures and sources of natural medicinal ingredients for skin pigmentation treatment through antioxidant stress and inhibition of melanin synthesis.

Pharmacological studies have revealed the potential of numerous natural products in alleviating skin pigmentation [2]. On account of their mild and natural properties, natural products are considered effective alternatives to chemically synthesized whitening and spot-lightening products. Consequently, they have emerged as a prominent subject of interest in both cosmetic and pharmaceutical research, garnering increasing favor among consumers [33]. Based on differences in pharmacological activities, natural medicinal ingredients suitable for addressing skin pigmentation can be categorized as tyrosinase inhibitors, antioxidants, and multi-pathway therapeutic agents (Figure 2 and Table 1) [26].

#### 3.1.1. Multi-Pathway Therapeutic Agents

1. Arbutin

Arbutin (ARB) is a hydroquinone glucoside found in many plants and widely used as a natural whitening agent. For natural ARB, it includes two subtypes, namely α-arbutin and β-arbutin. Among them, α-arbutin exhibits a stronger whitening effect and reduces pigmentation by competitively inhibiting tyrosinase activity [34,35]. Additionally, ARB plays a role in melanin degradation and transfer processes [36]. It can reduce the size of melanosomes and impede the transfer of melanin from melanocytes to adjacent keratinocytes, thereby preventing excessive melanin dissemination to the outermost layers of the epidermis [37]. Previous studies have demonstrated that ARB with a moderate dose (0.25 g/100 g) can significantly reduce tyrosinase activity, increase skin superoxide dismutase (SOD) activity, and reduce the production of free radical, thus relieving skin pigmentation diseases [38].

2. Azelaic Acid

Azelaic acid (AZA) is a naturally occurring saturated dicarboxylic acid, presenting as white to slightly yellowish monoclinic prisms, needle-shaped crystals, or powder. It is recognized for its potent antibacterial, anti-inflammatory, and antioxidant properties. It is commonly employed in treating acne, melasma, seborrheic dermatitis, and melanoma. Notably, AZA has obtained approval from the US FDA for papulopustular acne treatment [39]. Research indicated that AZA competitively inhibits tyrosinase activity and disrupts melanocyte function by impeding DNA synthesis and mitochondrial enzymes [40]. AZA also exhibits apparent anti-inflammatory properties to mitigate inflammation associated with pigmentation [41]. Moreover, AZA normalizes aberrant keratinization processes, regulates melanin accumulation through the turnover of skin cells, and promotes their normal growth [42]. However, owing to its poor solubility and permeability, AZA typically needs to be incorporated at high concentrations in drug formulations. Currently, the sodium hyaluronate nanoemulsions loaded with AZA have been developed to surmount the skin barrier and enhance skin retention rates, thereby improving the therapeutic efficacy [43].

3. Aloesin

Aloe curacao is a perennial evergreen herb belonging to the genus Aloe in the lily family, and it has been utilized for numerous years in the treatment of various skin conditions such as eczema, burn wounds, skin infections, and common acne [44,45]. Aloe curacao comprises a variety of chromone compounds, with aloesin, a natural hydroxymethyl chromone compound, being the primary tyrosinase inhibitory component in aloe curacao. It is a white needle-like crystal, soluble in organic solvents such as methanol, ethanol, DMSO, etc. Aloesin can competitively and non-competitively inhibit tyrosine hydroxylase activity, as well as competitively inhibit Dihydroxyphenylalanine (DOPA) oxidation, with its effect being significantly more potent than that of β-arbutin [46,47].

4. Glabridin

Glabridin (GLA) is an isoflavone isomer extracted from licorice root. It is often referred to in conjunction with glycyrrhizin and glycyrrhizin sweeteners, the latter being a glycoside composed of glycyrrhizinic acid and two molecules of glucuronic acid. Glabridin itself is an odorless white to slightly yellow crystalline powder that possesses a distinct sweet taste. GLA shows a wide range of biological activities, including anti-inflammatory, antioxidant, antitumor, antibacterial, bone protective, cardiovascular protective, neuroprotective, hepatoprotective, anti-obesity, and antidiabetic effects [48]. It holds significant promise in drug development and medicine. GLA functions by inhibiting tyrosinase activity and disrupting the pigment production pathway, thereby mitigating UVB-induced pigmentation [49]. Nonetheless, the poor solubility and low bioavailability of GLA restrict its clinical utility. Several effective strategies have been developed to address this limitation. One such strategy involves combining the potent tyrosinase inhibitor GLA with the antioxidant resveratrol to form micellar solutions that enhance drug permeability and complexes that more effectively inhibit tyrosinase activity [50].

5. Auraptene

Auraptene (AUR) belongs to the Rutaceae and Apiaceae families and is a biologically active coumarin antioxidant [51]. Researchers have confirmed its moderate antioxidant activity through assays such as NO scavenging activity, ferric thiocyanate, and thiobarbituric acid. Furthermore, cell-based antioxidant assessments have validated auraptene’s antioxidant activity by up-regulation of antioxidant-related genes, including superoxide dismutase, catalase, and glutathione peroxidase, in human foreskin fibroblasts (HFF). AUR has also demonstrated anti-melanogenic activity by directly inhibiting tyrosinase and modulating the expression of major melanogenesis-related genes, including tyrosinase, TRP-1, and dopachrome interconversion isomerase, in mouse melanoma cell lines [52].

6. Resveratrol

Resveratrol (RES) is a stilbene, and a natural polyphenol found abundantly in sources such as grapes, blueberries, strawberries, and peanuts [53]. It appears as a white to light yellow powder that is odorless and insoluble in water. However, resveratrol is soluble in organic solvents such as ether, trichloromethane, methanol, ethanol, acetone, and ethyl acetate. Its notable anti-inflammatory and antioxidant effects are key factors driving its widespread use in cosmetics. RES has also presented the ability to modulate tyrosinase activity, reducing the expression of melanogenesis-related proteins, including tyrosinase, TRP, and MITF in melanoma cells [54]. In vivo studies have shown that topical application of resveratrol significantly reduces pigmentation on ultraviolet B (UVB)-stimulated guinea pig skin.

#### 3.1.2. Tyrosinase Inhibitors

1. Gedunin

Gedunin (GED), primarily found in the exocarp of *Azadirachta indica* and citrus fruits, exhibits a variety of biological activities [55]. GED reduces melanin synthesis stimulated by α-MSH and inhibits both tyrosinase activity and protein content. This effect was validated in a zebrafish melanogenesis model in vivo, where GED reduced the occurrence of pigmentation spots and melanin co-production in zebrafish embryonic, leading to a significant decrease in overall embryonic pigmentation. Additionally, increasing doses of GED resulted in reduced melanin content and mRNA levels of associated genes [56].

2. Calycosin

Calycosin (CA) is an isoflavone derived from *Astragalus*, a traditional Chinese medicinal herb known for its various pharmacological effects [57]. It is a white to light yellow to light orange powder, soluble in methanol. CA exhibits significant inhibitory effects on zebrafish pigmentation, with its IC50 being lower than that of hydroquinone, tretinoin, and arbutin. Besides, mullein isoflavones demonstrate a high binding affinity to the active site of tyrosinase [58].

3. Patuletin

Patuletin (PN) is the main flavonoid component in *Inula japonica* and primarily exhibits pharmacological activities such as anti-inflammatory, anti-tumor, anti-atherosclerotic, anti-melanogenic, analgesic, anti-aging, and anti-allergic effects [59]. IkSoo Lee et al. investigated the anti-melanin effect of PN on mouse melanoma cells (B16-F10) and zebrafish embryos. PN dose-dependently decreased melanocyte-stimulating hormone-induced melanogenesis and L-DOPA oxidation in B16-F10 cells. Furthermore, it reduced tyrosinase expression in a dose-dependent manner [60].

4. Curcumin

Curcumin (CUR), dimethoxycurcumin (DMC), and bisdemethoxycurcumin (BDMC) are the active polyphenolic compounds found in turmeric, a plant belonging to the ginger family *Curcuma longa*, collectively known as curcuminoids. CUR is a rare pigment with diketone structures found in the plant kingdom. It is classified as a diketone and is insoluble in water and ether. However, it is soluble in ethanol and glacial acetic acid. These curcuminoids exhibit a wide range of biological activities, including antioxidant, anti-inflammatory, antibacterial, antifungal, and anticancer effects [61]. In a specific study, researchers utilized zebrafish embryos and mouse melanoma cells (B16F10) to evaluate the anti-melanogenic activity of CUR and its two derivatives, DMC and BDMC. The study found that CUR and BDMC reduced α-MSH-induced melanogenesis in B16F10 cells, concurrently downregulating the expression of melanogenesis-related genes, such as TYR, MITF, TRP-1, and TRP-2 [62].

5. Pulsae

Pulsae (PS), a potent antioxidant ingredient, is found in hibiscus petals [63]. Karunarathne investigated whether anthocyanins from two different petal colors (purple and white) of Pulsae (PS) and Paektanshim (PTS) inhibit melanin biosynthesis [64]. The results of the experiment showed that PS and PTS moderately down-regulated mushroom tyrosinase activity in vitro and significantly reduced extracellular and intracellular melanogenesis in B16F10 cells while also inhibiting α-MSH-induced expression of MITF and tyrosinase. Furthermore, PS and PTS attenuated the pigmentation of α-MSH-stimulated zebrafish larvae [65].

6. Sour jujube kernel

Sour jujube kernel (SJK) is an important medicinal and food ingredient with dual properties in herbal medicine and traditional food [66]. SJK extract exhibits numerous biological effects, including antioxidant, sedative-hypnotic, anti-inflammatory, and anticancer activity, particularly in melanoma cancer [67]. Jujubesaponin B (JUB), the active ingredient in Jujubes acidum extract, demonstrates high antioxidant and anticancer capacity in melanoma cells. Molagoda showed that JUB effectively inhibits α-melanocyte-stimulating hormone (α-MSH)-induced melanogenesis and prevents pigmentation in zebrafish larvae.

#### 3.1.3. Antioxidants

1. Pterostilbene

Pterostilbene (PT) is a natural polyphenol found in herbaceous plants, including blueberry, *Dalbergia hupeana*, and *Ormosia hosiei*. PT is a diphenylethylene derivative that is soluble in hot methanol and dimethyl sulfoxide (DMSO) but insoluble in water. It shares a structural similarity with resveratrol (RES) [68]. The presence of two methoxyl groups in PT enhances its bioavailability and pharmacological potency. PT has been proven to protect human keratin-forming cells from UV radiation-induced photodamage through the antioxidant NRF2/ARE pathway [69]. In addition, PT can downregulate tyrosinase protein expression and demonstrate superior performance compared to resveratrol and resveratrol trimethyl ether in α-MSH-induced melanogenesis in mouse melanoma cells (B16-F10).

2. Ferulic acid

Ferulic acid (FA) is a phenolic acid component widely distributed in nature, predominantly found in Chinese herbs such as *Angelica sinensis*, Rhizoma Ligustici Chuanxiong, and Shengma. FA is a common aromatic acid found in the plant kingdom and is a component of suberin. It is rarely present in its free form in plants, primarily existing in a bound state with oligosaccharides, polyamines, lipids, and polysaccharides. FA exhibits a broad spectrum of biological activities and low toxicity, rendering it an excellent antioxidant [70]. In recent years, numerous studies have demonstrated its potent scavenging effect on reactive oxygen species, its ability to inhibit the elevation of intracellular ROS levels, and its antioxidant effects through involvement in multiple signaling pathways [71]. FA demonstrates superior skin permeability in vitro, and its topical application alone or in combination with other active ingredients has been shown to prevent oxidative damage effectively and attenuate UV-induced skin pigmentation [70].

3. Salidroside

Salidroside *Rhodiola rosea* glycoside is a bioactive component isolated from *Rhodiola rosea* with strong antioxidant, antiviral, neuroprotective, and hepatoprotective effects. Salidroside rhodiola rosea was able to down-regulate the expression of NOX2 in cells [72], showed similar efficacy in terms of cytotoxicity and inhibition of melanin synthesis compared to the arbutin-treated group, and showed stronger antioxidant efficacy in B16F10 cells [73].

4. Gallic acid

Gallic acid (3, 4, 5-trihydroxybenzoic acid) is a natural plant polyphenol antioxidant extracted from natural plants such as *Pentaphyllum officinale*, grapes, tea, etc., which possesses a wide range of biological activities such as antioxidant, anticancer, and anti-inflammatory. Gallic acid is a white to light brown needle-shaped crystal or powder. It is soluble in hot water, ether, ethanol, acetone, and glycerin but is poorly soluble in cold water. It is insoluble in benzene and chloroform [74]. The effects of gallic acid on melanogenesis and its molecular mechanisms were investigated using an in vitro model of B16F10 melanocytes, and it was found that gallic acid significantly inhibited melanin synthesis in a dose-dependent manner and decreased the expression of melanogenesis-related proteins, such as MITF and doppler pigment interconvertase [75].

5. Thymoquinone

Thymoquinone (TQ), the main active ingredient of *Nigella sativa* seeds (*Nigella cuminata*), has anticancer and chemosensitizing properties. Thymoquinone has a wide range of beneficial biological and pharmacological properties [76]. In addition to its immunomodulatory activity, it also has outstanding antioxidant, anti-inflammatory, anticancer, cardiovascular, and hepatoprotective activities. Thymoquinone acts by inducing cytoprotective enzymes, thereby protecting cells from oxidative stress-induced cellular damage. Several studies have reported that TQ upregulates the mRNA expression and activation of antioxidant cytoprotective enzymes, including catalase, superoxide dismutase, glutathione reductase, colloidal gold, and glutathione peroxidase, which function in the scavenging of hydrogen peroxide and superoxide radicals to prevent lipid peroxidation [77,78]. Therefore, TQ can exert therapeutic effects on pigmentation through its excellent antioxidant properties [78].

**Table 1:** Natural medicinal ingredients for skin pigmentation treatment.

Function	Ingredient	Mechanism	In Vitro/In Vivo Studies	References
Multi-pathway agents	Arbutin	Inhibition of tyrosinase activity; Enhancement of SOD enzyme activity	Melasma guinea pig model	[36,37,38]
	Azelaic acid	Anti-inflammatory; Inhibition of tyrosinase activity	B16F10 cell line	[39,40]
	Aloesin	Antioxidant; Inhibition of tyrosinase activity	In vitro Mushroom tyrosinase assay; B16F10 cell line	[46]
	Glabridin	Antioxidant; Inhibition of tyrosinase activity	In vitro Mushroom tyrosinase assay; B16F10 cell line	[48,49]
	Resveratrol	Antioxidant; Inhibition of tyrosinase activity	Guinea pig model	[54]
	Auraptene	Inhibition of tyrosinase activity; Antioxidant	HFF cell line; B16F10 cell line	[51]
Tyrosinase inhibitors	Gedunin	Inhibition of tyrosinase activity and protein amounts	B16F10 cell line; Zebrafish embryo model	[56]
	Calycosin	Inhibition of tyrosinase activity	Molecular docking technology; Zebrafish embryo model	[58]
	Patuletin	Reduced tyrosinase expression	B16F10 cell line; Zebrafish embryo model	[60]
	Curcumin	Inhibition of tyrosinase-related gene expression	B16F10 cell line; Zebrafish embryo model	[62]
	Pulsae	Inhibition of tyrosinase activity	In vitro Mushroom tyrosinase assay; B16F10 cell line	[65]
	Jujube flavonoids	Inhibition of MITF and tyrosinase	Mushroom tyrosinase assay; B16F10 cell line; Zebrafish embryo model	[65]
Antioxidants	Pterostilbene	Antioxidant; Inhibition of NRF2/ARE signaling pathway	B16F10 cell line; Keratin-forming cell line; Zebrafish embryo model	[68,69]
	Ferulic acid	Antioxidant	B16F10 cell line	[70,71]
	Salidroside	Antioxidant	B16F10 cell line; Guinea pig model	[73]
	Gallic acid	Antioxidant	B16F10 cell line	[75]
	Thymoquinone	Antioxidant	Swiss albino mice	[78]

### 3.2. Natural Extracts

Over a lengthy period of clinical practice, researchers have collected a great deal of knowledge in treating pigmentation with natural medicinal extracts [6]. Most natural whitening ingredients are processed and extracted using various solvents and function primarily by inhibiting tyrosinase activity to reduce melanin production or through antioxidant actions to mitigate skin pigmentation resulting from oxidative stress [79]. However, these extracts generally act through a combination of multiple ingredients rather than a single active component. The pharmacological mechanisms of these extracts remain underexplored and require further investigation using advanced molecular biology techniques.

**Figure 3:**
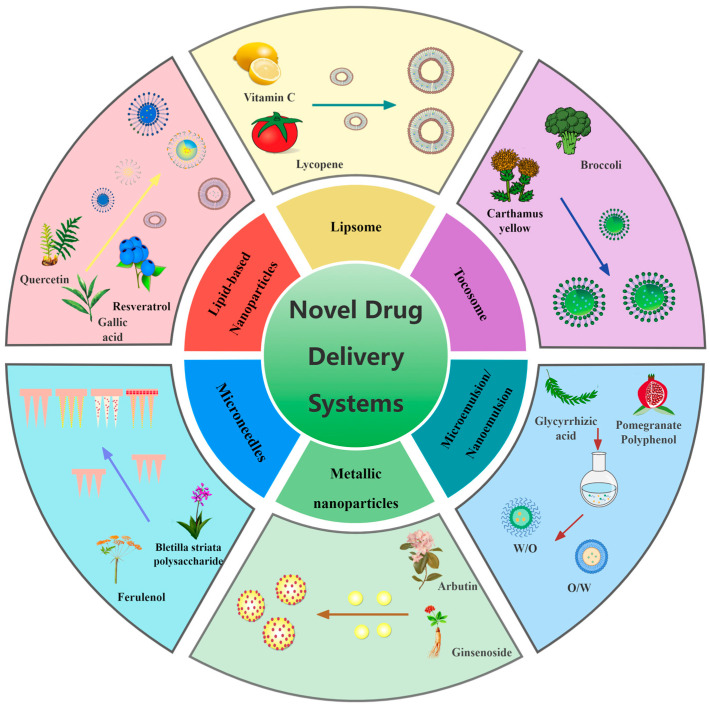
Novel drug delivery system for skin pigmentation treatment.

At present, many different types of natural products have been developed or have the potential for development [80], among which, coumaric acid, cinnamic acid, ferulic acid, coumarin, and other components of phenylpropanoids have strong inhibitory activity on human tyrosinase [81]. Aloe-emodin and rhodopsin in anthraquinones [82] have inhibitory effects on mushroom tyrosinase, mouse tyrosinase, and human tyrosinase. Polysaccharide components such as Ganoderma lucidum polysaccharides, Bletilla striata polysaccharides, Poria polysaccharides have antioxidant effects [83,84,85,86], and the mechanism of antioxidant occurrence of polysaccharides from the cellular level can be broadly categorized into scavenging free radicals, up-regulation of antioxidant enzyme activity, reduction of lipid metabolites, protection of organelles in the cell and modulation of the cellular signaling pathway to inhibit cell apoptosis [87]. Flavonoids include flavonols, isoflavones, flavan-3-ols, flavanones, and chalcones, etc., with various mechanisms of action; they have antioxidant and tyrosinase inhibitory activities and are also good ultraviolet absorbers [88,89]. Curcumin, rosemarinic acid, tea polyphenols, and other components of polyphenols [90,91,92] can play an antioxidant and anti-aging role by scavenging hydroxyl radicals, enhancing SOD in the body, and improving peroxidase activity. The details are shown in Table 2 and Figure 3.

**Table 2:** Natural extracts for skin pigmentation treatment.

Extract	Source	In Vitro/In Vivo Studies	Mechanism	References
Polysaccharides	Ganoderma	Zebrafish embryo model; Guinea pig model	Antagonism of cAMP/PKA and ROS/MAPK signaling pathways	[85]
	Morchella esculenta	B16F10 cell line; Zebrafish embryo model	Dose-dependent inhibition of tyrosinase activity and reduction of MITF and TRPs protein expression	[86]
	Bletilla striata	In vitro free radical scavenging ability test	Antioxidant	[83]
	Poria	Mushroom tyrosinase assay	Inhibition of tyrosinase activity	[84]
	Esenticosus	In vitro free radical scavenging ability test	Antioxidant	[86]
Flavonoids	Selaginella	Mushroom tyrosinase assay; B16F10 cell line, Zebrafish embryo model	Antioxidant; Inhibition of tyrosinase, MAPK, and MITF pathway expression	[93]
	Theaflavin	Spectral analysis; Molecular docking; Zebrafish embryo model	Antioxidant; Inhibition of tyrosinase activity	[92]
	Hesperidin	Mushroom tyrosinase assay; B16F10 cell line	Activation of the MEK/ErK1/2 pathway	[94,95]
	Tanshinone	HEM cell line	Activation of Nrf2 antioxidant pathway	[94,96]
	Ginkgo leaves	In vitro free radical scavenging ability test	Antioxidant	[97]
Polyphenols	Tea polyphenols	B16F10 cell line; Zebrafish embryo model	Inhibition of tyrosinase activity	[98]
	Ginseng phenolic acid	B16F10 cell line; Zebrafish embryo model	Inhibition of melanin synthase through the cAMP/PKA signaling pathway	[91]
	Brown algae	B16F10 cell line; Zebrafish embryo model	Inhibition of tyrosinase activity and regulation of protein expression of the MITF/CREB signaling pathway	[99]
	Pomegranate	In vitro free radical scavenging ability test	Antioxidant	[100]
	Orange peel	In vitro free radical scavenging ability test	Antioxidant	[101]
Other natural product extracts	CalendulaofficinalisL	Mushroom tyrosinase assay	Inhibition of tyrosinase activity	[102]
	Kava pepper	B16F10 cell line	Regulation of tyrosinase and MITF activity	[103]
	Edible mushrooms	Zebrafish embryo model	Dose-dependent inhibition of melanogenesis	[104]
	Olive leaves	Zebrafish embryo model	Inhibition of tyrosinase activity	[105]
	Coix seed bran oil	B16F10 cell line; Zebrafish embryo model	Inhibition of tyrosinase activity	[106]
	Rice extracts	B16 cell line; Zebrafish embryo model	Antioxidant; Regulation of tyrosinase; Regulation MITF activity	[107]

## 4. Novel Drug Delivery System for Skin Pigmentation Treatment

Natural medicines have demonstrated significant potential in the treatment of skin pigmentation, garnering increasing interest from researchers [6]. The effectiveness of a natural product is influenced by its time to reach the target site and its concentration at the site of action. The low solubility and high dosage requirements of herbal components, coupled with poor patient compliance, necessitate improved drug delivery methods for their potential application [108]. To enhance the efficacy of natural ingredients in treating skin pigmentation, optimize therapeutic outcomes, improve patient compliance, and minimize adverse effects, phytochemicals are often formulated into nanosized particles or incorporated into nanostructures. Nano-formulations offer several advantages over free drug molecules, including increased solubility, improved pharmacokinetics, enhanced efficacy, and reduced toxicity [109]. Various innovative drug delivery systems are being developed for pigmentation therapy, including lipid-based nanoparticles, microemulsions, nanoemulsions, metal nanoparticles, tocosomes, and microneedles, as illustrated in Figure 3 and detailed in Table 3. These novel formulations have shown substantial benefits in enhancing drug bioavailability and reducing drug toxicity [110].

### 4.1. Lipsome

Liposomes, composed of phospholipids and cholesterol, exhibit strong drug protection and targeting capabilities [111]. The release of drugs from liposomes is influenced by the liposome composition, the permeability of the bilayer, and the nature of the encapsulated or loaded drugs. Additionally, drug release can result from phase transitions of lipids triggered by external stimuli such as changes in temperature or pH [112]. Liposomes protect drugs by providing greater control over drug release and preventing interactions with enzymes in the body [113]. For example, a study investigated the effectiveness of liposome-mediated delivery for drugs that extend the lifespan of Caenorhabditis elegans. It was found that liposome-encapsulated glutathione extended lifespan by increasing infection resistance and exhibited anti-aging effects [114]. However, liposomes face limitations in penetrating the stratum corneum, which restricts their use in skin drug delivery. Additional disadvantages include poor encapsulation of hydrophilic drugs and inadequate storage stability due to drug leakage in the medium [115,116]. These shortcomings of conventional liposomes have prompted the development of new lipid nanoparticles, such as solid lipid nanoparticles, nanostructured lipid carriers, and delivery bodies [110].

### 4.2. Lipid-Based Nanoparticles

Lipid-based nanoparticles offer significant advantages for encapsulating and delivering various bioactive compounds. Based on the preparation methods and physicochemical properties of the formulations, lipid nanoparticles are classified into five categories: liposomes, nonionic surfactant vesicles (niosomes), transfersomes, solid lipid nanoparticles (SLNs), and nanostructured lipid carriers (NLCs) [110].

#### 4.2.1. Solid Lipid Nanoparticles

Solid lipid nanoparticles (SLNs) were introduced over 30 years ago, and extensive research has established their efficacy and advantages over emulsions, micelles, polymer nanoparticles, and liposomes. SLNs are constructed from lipids that share similar attributes: incorporation of surfactants/cosurfactants, stability across various temperatures, and a low melting point [117]. SLNs serve as carrier materials utilizing natural or synthetic solid lipids. Techniques such as thin film-ultrasonic dispersion, emulsion-evaporation-low temperature curing, and high-pressure homogenization are employed to fabricate SLN by encapsulating or embedding physiologically compatible drugs within lipid-like cores [13]. SLNs are capable of encapsulating drugs, thereby enabling their existence as nanoparticles within an aqueous solution. This process significantly enhances the solubility of hydrophobic drugs, making them more stable than in pure water conditions [118]. Moreover, SLNs can create a continuous film on the skin surface, which hydrates the stratum corneum, thereby increasing drug permeability and enhancing bioavailability [2].

Self-assembly of natural polymers into highly efficient and ordered lignin nanoparticles (LNPs) through a high-pressure homogenization method can improve their solubility and exhibit better UV shielding and antioxidant properties [119]. Similarly, quercetin-loaded PLGA-TPGS nanoparticles (PLGA-TPGSNPs) overcome these limitations by enhancing quercetin’s hydrophilicity and anti-UVB efficacy. Additionally, it was observed that PLGA-TPGSNPs mitigated UVB-induced macroscopic and histopathological changes in mice’s skin, indicating that the copolymer may act as an effective nanocarrier for treating skin damage and associated disorders [120,121].

#### 4.2.2. Nanostructured Lipid Carriers

Following the development of solid lipid nanoparticles (SLNs), nanostructured lipid carriers (NLCs) were introduced to improve drug encapsulation and prevent drug leakage. NLCs are widely used as drug delivery systems for lipophilic drugs [122]. Compared to SLNs, NLCs offer enhanced drug-loading capacity and sustained release capabilities [123]. NLCs are characterized by an unstructured lipid core and a monolayer of surfactants at the periphery. The core comprises a mixture of solid and liquid lipids, which forms an imperfect crystalline structure that facilitates increased drug loading. In contrast, SLNs exhibit a saturated drug-loading capacity due to their solid lattice. Furthermore, the liquid phase in NLCs helps to prevent drug release during storage [124]. Utilizing NLCs to encapsulate poorly water-soluble resveratrol can significantly reduce drug particle size while displaying good water dispersibility. The lipophilic resveratrol components released from NLCs maintain their nanoscale size, showcasing excellent physiological stability and targeting properties [125]. Additionally, they exhibit higher antioxidant activity than raw resveratrol material. Arbutin, due to its hydrophilicity, exhibits poor skin permeability and a low utilization rate for topical application. Radmard utilized the ultrasonic method to encapsulate arbutin in NLCs, thereby preparing an environmentally friendly green preparation, which increased the skin permeability and storage stability of arbutin [126].

#### 4.2.3. Transferosomes

Transfersomes are an advanced form of liposomes composed of phosphatidylcholine and an edge activator. They represent a specialized type of nanocarrier designed for effective drug delivery to the skin. Transfersomes differ from conventional liposomes in that they possess more flexible bilayer membranes, which enables these carriers to penetrate deeply into tissues or reach specific active sites [127]. Being inherently amphiphilic, transferosomes can simultaneously transport hydrophilic and hydrophobic drugs across the skin barrier, depending on the composition and dosage of the drug [128]. In a study conducted by Avadhani, lipid transferosomes incorporating hyaluronic acid (HA) and epigallocatechin-3-gallate (EGCG) were prepared using a thin-film hydration approach with soy phosphatidylcholine and sodium cholate as raw materials. Compared to the raw material, the enhanced formulation of EGCG and HA transferosomes exhibited significantly improved skin penetration and EGCG deposition. Furthermore, the synergistic UV protection ability of both compounds was augmented, and these transferosomes also exhibited antioxidant and anti-aging effects, rendering them potentially valuable for applications in sunscreens or emulsions [129].

### 4.3. Microemulsions/Nanoemulsions

In recent years, various methods of preparing nanoemulsions and microemulsions have been validated as means to enhance the bioavailability and solubility of oily components [130]. Microemulsions and nanoemulsions are similar in many respects. Both are systems that exhibit a milky or translucent appearance due to their small particle size and low viscosity, with the drug either dispersed or adsorbed within the inner phase of the droplets. A microemulsion is a low-viscosity, transparent or translucent, optically isotropic, and thermodynamically stable system spontaneously formed by mixing water, oil, and surfactant in appropriate proportions [131]. The average particle size in a microemulsion ranges from approximately 100 to 400 nm. In contrast, a nanoemulsion is a thermodynamically unstable system characterized by droplets with an average particle size between 0 and 100 nm [132]. Due to their extremely small droplet size, nanoemulsions are not influenced by gravity but are affected by Brownian motion, which helps mitigate or delay traditional instability issues. Although nanoemulsions are thermodynamically unstable, they are kinetically stable, which enhances their resistance to degradation. These properties make nanoemulsions particularly advantageous for drug preservation [133].

Yan et al. prepared glycyrrhizic acid (GA) ionic liquid microemulsion (IL-ME) (GA-IL-ME). In vitro, transdermal experiments demonstrated that the cumulative permeation amount of GA-IL-ME per unit area within 24 h was 1.34 times greater than that of the traditional oil-in-water (O/W) type microemulsion containing glycyrrhetinic acid. Therefore, the newly prepared ionic liquid microemulsion combines the advantages of both microemulsions and ionic liquids, resulting in significantly increased solubility of the water-soluble medication glycyrrhetinic acid, enhanced permeation effects, and improved therapeutic efficacy [14].

### 4.4. Metallic Nanoparticles

Metallic nanoparticles are distinguished drug carrier materials characterized by their flexible and controllable shape, size, and surface chemistry. They are bioinert, non-toxic, and possess excellent biocompatibility [134]. Natural products serve as reducing agents in combination with metals such as gold, silver, and copper to prepare metallic nanoparticles. This collaborative strategy not only reduces the reliance on harmful chemicals but also enhances bioavailability [135]. Arbutin, a natural polyphenol with potent reducing properties, is an ideal bioactive ingredient for the synthesis of gold nanoparticles (GNPs). A study combined ursolic acid with gold nanoparticles using an eco-friendly synthesis approach. Experimental results indicated that GNP-A possesses enhanced skin-whitening properties compared to ursolic acid alone, and it significantly increases anti-melanogenesis activity [136]. Zuly utilized ginseng berry extracts (GBE) as reducing and stabilizing agents to synthesize multifunctional gold nanoparticles (AuNPs) and silver nanoparticles (AgNPs) through green chemistry approaches. The findings demonstrated that the phytochemicals in GBE effectively reduced and encapsulated gold and silver ions, resulting in GBAuNPs and GBAgNPs with outstanding antibacterial, antioxidant, and tyrosinase inhibition capabilities. Consequently, GBE demonstrates potential as an antibacterial agent, antioxidant, and skin protectant [137,138].

### 4.5. Microneedles

With the emergence of microelectronic processing technology, the use of microneedles as a drug delivery system has gained popularity, demonstrating its prominent advantages in the field of transdermal delivery. Microneedles, due to their small size, are capable of penetrating the stratum corneum barrier of the skin, facilitating drug permeation and allowing for targeted drug delivery [139]. Moreover, they do not stimulate the sensory nerves in the dermis layer, thus avoiding any pain or discomfort. Microneedles are commonly classified into solid microneedles, coated microneedles, hollow microneedles, dissolving microneedles, and hydrogel microneedles [140]. With the increasing research on natural medicines, soluble microneedles are gradually used for the delivery of natural active ingredients, which greatly improve the bioavailability of natural products [141]. At the same time, relying on the characteristics of microneedles that are painless and minimally invasive can improve the patient’s compliance during the treatment process.

Ferulic acid is easily absorbed when taken orally; it is rapidly metabolized and has a short half-life [142]. However, when formulated into microneedles containing ferulic acid, its transdermal permeation is significantly increased, leading to an enhanced anti-inflammatory and antioxidant effect [143]. To create a unique dissolvable microneedle formulation, our laboratory loaded tranexamic acid and licorice extract into polyvinyl alcohol (PVA) and polyvinylpyrrolidone (PVP) matrix materials. From the experimental results, it can be found that the dissolvable microneedle formulation significantly improved drug permeation. With this microneedle formulation, tranexamic acid’s bioavailability was markedly expanded, and the signs of hyperpigmentation in a guinea pig model of melasma were significantly diminished [15].

### 4.6. Tocosome

Tocosomes are vesicles primarily composed of tocopherol and tocopherol acyl phosphate molecules. Tocopherol is used as an antioxidant and skin conditioner in the cosmetic industry, and it exerts its antioxidant effects by scavenging peroxyl free radicals. The term “Tocosomes” refers to long-term stable nanovesicles. Novel nanocarrier systems, both with and without cholesterol, containing α-tocopheryl phosphate (TP) and di-α-tocopheryl phosphate (T2P), as well as various lipids and phospholipids, were prepared using the Mozafari method [144]. This method does not involve potentially toxic solvents, detergents, or harsh treatments such as ultrasound or high-shear homogenization. Studies have demonstrated the efficient simultaneous transport of fat-soluble vitamin E/tocopherol and water-soluble vitamin C/glutathione, enhancing the synergistic effects of different drugs in skin pigmentation treatments [145].

**Table 3:** Novel transdermal drug delivery system for skin pigmentation treatment.

Formulation	Ingredient	Method/Material	Enhancement	Reference
Lipsome	Glutathione	Liposome extruder purification	Increase medication uptake	[114]
	Lycopene	Thin film hydration method	Skin permeability and antioxidation	[146]
Solid lipid nanoparticles	Lignin	High-pressure homogenization	UV shielding effect and antioxidation	[119]
	Quercetin	Nanoprecipitation	Hydrophilicity	[121]
	Auraptene	Hot homogenization and ultrasonication	Skin permeability	[51]
Nanostructured lipid carrier	Resveratrol	Ultrasonication	Targeting and antioxidant activity	[125]
	Arbutin	Ultrasonication	Skin permeability and stability	[126]
Transferosomes	Epigallocatechin-3-gallate	High-pressure homogenization	Skin permeability and antioxidation	[129]
Microemulsions	Glycyrrhizic acid	Ionic liquid microemulsion	Solubility and permeability	[14]
Nanoemulsions	Pomegranate peel	Pomegranate seed oil	Skin permeation	[147]
Metallic nanoparticles	Arbutin	Eco-friendly synthesis	Antimelanogenic activity	[136]
	Ginseng berry	Eco-friendly synthesis	Antibacterial and antioxidant activity	[138]
Microneedles	Ferulic acid	Solid microneedles	Skin permeability	[142]
	Tranexamic acid and licorice extract	PVA and PVP	Bioavailability	[15]
	Resveratrol	Acrylic resin E100/PVP-K90	Stability	[148]
	Glabridin	Cyclodextrin	Transdermal penetration and retention time	[149]
	Arbutin	HPMC and PVP	Skin permeability	[150]
Tocosome	vitamin C/glutathione	Mozafari method	Improve transshipment efficiency	[145]

## 5. Evaluation Methods for Pigmentation Treatment

**Figure 4:**
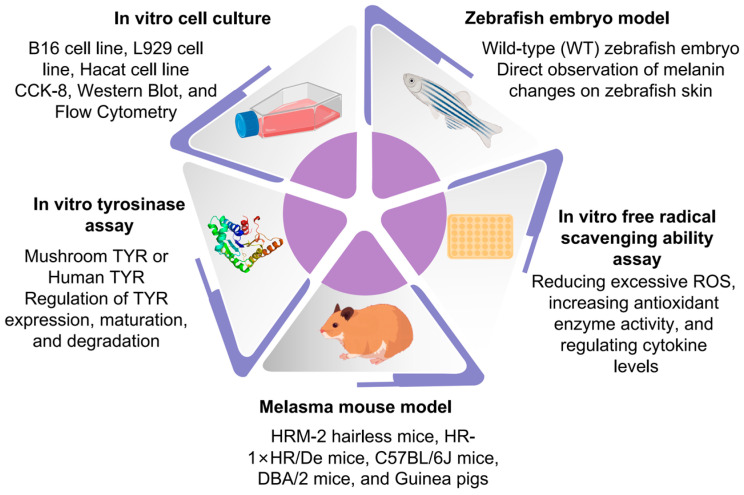
Current evaluation indicators of natural products for the treatment of hyperpigmentation.

At present, there is no universal standard for the evaluation of whitening and spot-lightening products. In clinical research, alterations in the extent of skin pigmentation before and after pharmacological intervention are frequently utilized as evaluation metrics. However, the subjective nature of these observations and evaluations may undermine the reliability of the efficacy assessment [151]. Various models, both in vitro and in vivo, have been developed to investigate the inhibitory effects of treatments on hyperpigmentation processes. In vitro experimental designs focus on assessing the impact of tyrosinase inhibitors and antioxidants on melanin reduction. More sophisticated experimental setups involve the use of cultured melanocytes, fibroblasts, and keratinocyte cell lines, among others [152]. These studies aim to determine the influence of pharmacological agents on melanin synthesis, transfer, and various other cellular-level processes. Additionally, zebrafish embryo models, transgenic nude mouse models featuring human epidermis with melanin, and guinea pig melasma models currently serve as viable in vivo experimental platforms (Figure 4) [10,11,152]. While many natural substances have proven useful in in vitro studies, a limited number have progressed to clinical trial stages, continuing to demonstrate effectiveness. Hence, there is a growing imperative to identify diverse pharmacological agents for treating pigmentation-related disorders and to develop safer, more efficient comprehensive evaluation methods. Such advancements are pivotal for the future research, evaluation, and application of whitening and spot-removing formulations [153,154].

### 5.1. Tyrosinase Activity

Tyrosinase (TYR) is a key rate-limiting enzyme in the melanin biosynthesis pathway, and overproduction of melanin has been associated with abnormally high expression of TYR [155]. Consequently, tyrosinase has emerged as a primary target for many melanin treatment drugs, which aim to regulate the expression, maturation, and degradation of TYR or directly inhibit its catalytic activity [156,157]. With the increasing focus on natural product research, ongoing studies are primarily dedicated to the development and evaluation of various natural tyrosinase inhibitors [7]. The inhibitory action of enzyme inhibitors is reversible [158] and generally categorized into four types: irreversible inhibition, non-specific inhibition, competitive inhibition, and non-competitive inhibition [159].

Mushroom tyrosinase (mTYR) is frequently employed as an in vitro enzyme model for developing skin whitening treatments due to its easy purification, simplicity of use, and commercial availability. However, there are notable differences between mTYR and human tyrosinase (hTYR) in several aspects. The secretion form of mushroom tyrosinase is a tetrameric enzyme found in the cell cytoplasm, whereas human tyrosinase exists as a monomer in an inactive glycosylated membrane-bound form [160]. Moreover, studies indicate that human tyrosinase exhibits an L-DOPA oxidation activity affinity nearly six times higher than that of mushroom tyrosinase. The Km values for L-DOPA in humans and mushroom tyrosinase are 0.31 mM and 1.88 mM, respectively [161]. It has been observed that many melanin synthesis inhibitors demonstrate inhibitory effects on mushroom tyrosinase while exerting minimal inhibition on human tyrosinase. Several plant extracts or individual components are utilized as whitening ingredients in cosmetics. Apart from conducting mTYR inhibition tests, further experiments are needed to determine its therapeutic efficacy and drug screening using popular whitening agents such as tretinoin, arbutin, or hydroquinone as positive controls [28].

### 5.2. Antioxidant Capacity

The excessive accumulation of free radicals or ROS in the body leads to a state of oxidative stress [8,162]. Excessive levels of free radicals and ROS can interact with DNA and proteins in the body, causing oxidative damage and resulting in skin aging. Furthermore, free radicals and ROS can also act on the basal layer of the skin, activating tyrosinase, inducing melanocytes to promote melanin secretion, and leading to abnormal melanin metabolism, ultimately causing skin conditions associated with hyperpigmentation [29]. Therefore, repairing oxidative stress damage and restoring the oxidative balance in the body are also important points in the treatment of pigmentation diseases.

Antioxidants function by reducing or eliminating excessive levels of ROS that give rise to oxidative stress, enhancing the activity of antioxidant enzymes, and regulating the levels of relevant cytokines, among other mechanisms [163]. The evaluation of antioxidants is mainly conducted at three levels: in vitro, in cells, and in animals. In vitro evaluation methods use redox reactions to detect the scavenging effect of various antioxidants on free radicals, such as the 1,1-diphenyl-2-picrylhydrazyl (DPPH) assay, the 2,2′-azino-bis (3-ethylbenzothiazoline-6-sulfonic acid) (ABTS) assay, the superoxide anion radical scavenging (SRSA) assay, and the ferric ion reducing/antioxidant power (FRAP) assay [164]. The approaches based on the cellular level largely entail assessing antioxidant enzyme activity, peroxide generation, and ROS levels in the evaluation of antioxidant activity in vivo. In addition to these indicators, the assessment of antioxidant activity at the animal level can also be done by observing the survival time and phenotypes of animal models to determine the antioxidant efficacy of compounds. Caenorhabditis elegans, Drosophila melanogaster, and mice are often used as model organisms [165].

### 5.3. Cell Model

Cell model testing involves culturing melanocytes in vitro and assessing cell vitality and metabolic functions after exposure to the test substance. In vitro, cultured melanocytes can be used to determine tyrosinase activity and melanin content, which is the most commonly used method for studying whitening active substances [166]. These include the CCK-8 assay and colony formation assay, which are used to evaluate the effects of the test drugs on cell proliferation. The distribution of the cell cycle, cell apoptosis, and reactive oxygen species (ROS) levels can be examined using flow cytometry. The migration and invasion capabilities of cells can be assessed through Transwell migration/invasion assays. Western blotting is employed to detect the expression of relevant proteins [151,167].

In vitro assays, mouse melanoma B16 cells are often used for evaluation. B16 cells are derived from highly metastatic mouse skin melanoma, and their genomic composition exhibits a high similarity to human epidermal melanocytes [9]. Moreover, B16 cells, which have the same mechanism of melanogenesis as normal human melanocytes, are relatively easy to culture and maintain pigmentation, making them the preferred cell model for testing the biological effects of test substances on melanocytes. Additionally, cell culture requires stringent sterile conditions (temperature, CO_2_, serum, culture media, trypsin, etc.), and even established mouse melanoma cell lines are not always easy to culture [168].

### 5.4. Zebrafish Model

The zebrafish model has become a commonly used in vivo animal model. Compared to mammalian models, this type of model offers advantages such as low cost, small size, and ease of handling and maintenance [169]. This model can track and study the depigmentation activity of many bioactive compounds and is widely recognized as a good animal model [170]. Furthermore, zebrafish skin shares many similarities with human skin. Drugs have a higher penetration rate in zebrafish skin and gills, allowing for direct observation of melanin changes on the surface of zebrafish skin without complex experimental procedures. This makes it suitable for studying melanogenesis inhibitors [10]. Zebrafish have higher reproductive capacity than mice, making them highly adaptable for large-scale drug treatments and suitable for screening drugs and cosmetics. Additionally, the genetic mechanisms of pigmentation disorders can be rapidly investigated using gene modification experiments in numerous individual zebrafish [171].

In most cases, comparisons are made between wild-type (WT) zebrafish and other transgenic variants or mutants, with experiments starting during the embryonic stage 2–12 h after fertilization. In melanin inhibition assays, embryos are typically incubated in an aquatic medium with a pH of around 7 and under controlled environmental temperatures (25–30 °C) [170]. Since the embryos obtain nutrients from their yolks, no additional nutritional substances are added during the early stages of embryonic development. When studying melanin inhibition experiments using zebrafish embryo models, PTU is commonly employed as a control drug. PTU is an organic sulfur TYR inhibitor typically used to block zebrafish pigmentation by inhibiting TYR-dependent melanogenesis pathways without generating any adverse toxicity [171].

### 5.5. Mouse Model

Mice are the most often used experimental animals in animal models for studying pigmentation abnormalities like melasma. The mouse model is the only model that allows for the genetic continuum from physiological to molecular levels through cell lines [172]. When establishing animal models for melasma, the selection of experimental animals is influenced by animal strains, gender, and age and should be based on the specific experimental objectives. HRM-2 hairless mice, (HR-1 × HR/De)F1 mice, C57BL/6J mice, DBA/2 mice, and guinea pigs with brown-yellow fur are frequently utilized [11]. Among them, guinea pigs have a larger body size, providing sufficient skin tissue for experimental research. Moreover, the quantity and distribution of melanocytes and melanosomes in the brown-yellow skin epidermis of guinea pigs are similar to those in humans. Following ultraviolet (UV) irradiation, guinea pig melanocytes and melanosomes exhibit reactions similar to human skin pigmentation. Therefore, guinea pigs are commonly used in studies related to melasma [173]. Currently, the preparation of melasma animal models primarily involves simulating clinical factors that induce skin pigmentation. According to the possible pathogenic factors, commonly used modeling methods include UV-induced modeling, progesterone-induced modeling, and a combination of UV and progesterone-induced modeling [152]. The establishment of clinically representative and stable melasma animal models is an important tool for studying the pathogenesis of melasma, conducting research on natural product development, and exploring effective prevention and treatment strategies for melasma.

Although evaluation models can effectively simulate human diseases, the human genome, epigenome, and lifestyle are unique to each individual. The various evaluation methods mentioned above have respective limitations (Table 4), and the interactions between proteins in animal models and humans may differ. The established evaluation models cannot fully reflect the extensive genetic diversity in humans, as well as the specific mechanistic pathways and therapeutic effects of different types of natural product ingredients or formulations for human pigmentation disorders.

**Table 4:** Advantages and disadvantages of currently commonly used evaluation methods.

Evaluation Methods	Type/Strain	Advantage	Disadvantage	Reference
In vitro tyrosinase assay	mTYR/hTYR	Lower cost and shorter experimental period	Poor enzyme homology and large differences in active sites	[155,158]
In vitro free radical scavenging ability assay	DPPH/ABTS/SRSA/FRAP	Inexpensive, fast detection, simple operation	Differences in free radical scavenging effects between in vivo and vitro	[162,163]
In vitro cell culture assay	B16/A375 cell line	A shorter experimental period and the possibility to study intracellular mechanisms	Specific culture conditions	[9,151]
In vitro zebrafish embryo model testing	Wild type zebrafish	Easy to observe and capable of specific mechanistic studies	Specific culture conditions	[10,168,169]
In vivo melasma mouse model experiments	Brown female guinea pig	Continuum of lifeforms from molecules to genetics, high homology, and the possibility of specific mechanism studies	The long incubation period, following 3R principles and experimental ethics required	[11,171,173]

## 6. Conclusions and Future Discussions

Overall, developing effective skin pigmentation treatment agents from natural products offers higher safety and relatively fewer toxic and side effects compared to chemical synthetic substances. As people increasingly seek safer and more effective whitening agents, the demand for natural whitening substances is expected to rise significantly. Natural whitening substances can not only lighten and protect the skin but also contribute to its repair. However, they may produce negative effects in specific situations, with skin allergic reactions being the most common. Although modern topical formulations have introduced a variety of new dosage forms, their drug encapsulation efficacy often restricts them from delivering individual components, resulting in formulations that may be indistinguishable from chemically synthesized counterparts. To address this limitation, employing structurally modified multi-component carriers represents a potential solution [108,122].

Currently, various natural compound fractions and combinations are undergoing experimental testing, with some components showing promising results in the initial stages of clinical trials. Consequently, more clinical research is needed to design and develop novel, safe, and efficient whitening formulations based on natural products. Additionally, employing modern technologies and analytical methods to investigate the mechanisms of action of natural whitening products is a prominent current research trend. Specific analytical techniques include HPLC, MS, GC-MS, LC-MS, and FTIR, which are used to elucidate the composition of natural products. Concurrently, molecular techniques such as ELISA, Western blot, and qPCR are employed to study the in vivo mechanisms of these products. However, the complex compositions and multiple targets associated with natural whitening products present significant challenges in analyzing their active ingredients and mechanisms of action. Therefore, integrating modern technology and analytical methods to explore the mechanisms of action of natural whitening products, scientifically elucidating their usage and dosage, and investigating the biological basis of natural product formulations and disease represents a crucial problem that must be addressed in the development of natural product formulations for treating pigmentation disorders.

## Data Availability

No additional data are available.

## Abbreviations

TYR	tyrosinase
TRP-2	tyrosinase-related protein-2
TRP-1	tyrosinase-related protein-1
α-MSH	α-melanocyte stimulating hormone
MITF	microphthalmic aberrant transcription factor
ROS	reactive oxygen species
ARB	Arbutin
AZA	Azelaic Acid
DOPA	Dihydroxyphenylalanine
GLA	Glabridin
AUR	Auraptene
HFF	human foreskin fibroblasts
RES	Resveratrol
GED	Gedunin
CA	Calycosin
PN	Patuletin
CUR	Curcumin
PS	Pulsae
PTS	Paektanshim
SJK	Sour jujube kernel
JUB	Jujubesaponin B
PT	Pterostilbene
FA	Ferulic acid
TQ	Thymoquinone
GSH	Glutathione
SLNs	solid lipid nanoparticles
NLCs	nanostructured lipid carriers
LNPs	lignin nanoparticles
PLGA-TPGSNPs	PLGA-TPGS nanoparticles
AUR-SLNs	AUR-loaded solid lipid nanoparticles
HA	hyaluronic acid
EGCG	epigallocatechin-3-gallate
GA	glycyrrhizic acid
IL-ME	ionic liquid microemulsion
O/W	oil-in-water
AuNPs	gold nanoparticles
AgNPs	silver nanoparticles
PVA	polyvinyl alcohol
PVP	polyvinylpyrrolidone
TP	a-tocopheryl phosphate
T2P	di-a-tocopheryl phosphate
HPMC	hydroxypropyl methyl cellulose
mTYR	Mushroom tyrosinase
hTYR	human tyrosinase
L-DOPA	Levodopa
DPPH	1,1-diphenyl-2-picrylhydrazyl
ABTS	2,2′-azino-bis (3-ethylbenzothiazoline-6-sulfonic acid)
SRSA	superoxide anion radical scavenging
FRAP	ferric ion reducing/antioxidant power
B16	mouse melanoma cell line
A375	human melanoma cell line
WT	wild-type
